# Optimizing Nanoplasmonic Biosensor Sensitivity with Orientated Single Domain Antibodies

**DOI:** 10.1007/s11468-015-9969-3

**Published:** 2015-05-26

**Authors:** Marc P. Raphael, Joseph A. Christodoulides, Jeff M. Byers, George P. Anderson, Jinny L. Liu, Kendrick B. Turner, Ellen R. Goldman, James B. Delehanty

**Affiliations:** Bioelectronics and Sensing, Code 6363, Naval Research Laboratory, 4555 Overlook Ave SW, Washington, DC 20375 USA; Center for Bio/Molecular Science and Engineering, Code 6900, Naval Research Laboratory, 4555 Overlook Ave SW, Washington, DC 20375 USA

**Keywords:** LSPR, SPR, Single domain antibodies, Orientation, Ricin, Bio-threat

## Abstract

Localized surface plasmon resonance (LSPR) spectroscopy and imaging are emerging biosensor technologies which tout label-free biomolecule detection at the nanoscale and ease of integration with standard microscopy setups. The applicability of these techniques can be limited by the restrictions that surface-conjugated ligands must be both sufficiently small and orientated to meet analyte sensitivity requirements. We demonstrate that orientated single domain antibodies (sdAb) can optimize nanoplasmonic sensitivity by comparing three anti-ricin sdAb constructs to biotin-neutravidin, a model system for small and highly orientated ligand studies. LSPR imaging of electrostatically orientated sdAb exhibited a ricin sensitivity equivalent to that of the biotinylated LSPR biosensors for neutravidin. These results, combined with the facts that sdAb are highly stable and readily produced in bacteria and yeast, build a compelling case for the increased utilization of sdAbs in nanoplasmonic applications.

**Electronic supplementary material:**

The online version of this article (doi:10.1007/s11468-015-9969-3) contains supplementary material, which is available to authorized users.

## Introduction

Localized surface plasmon resonance (LSPR) biosensing is a label-free technique that enables biomolecule detection with nanoscale spatial resolution. Since its introduction, the technique has found a broad range of applications including studies of DNA-protein interactions [1], toxins [2], proteins [3, 4], and vesicles [5]. The small size of the individual sensors, typically 40 to 150 nm in diameter, has the potential to enable sensor miniaturization to a scale unapproachable by the closely related planar technique of surface plasmon resonance (SPR) biosensing. Also unique to LSPR is that the nanoplasmonic resonance condition is satisfied in a simple reflected or transmitted light geometry, common to both microscopy and spectroscopy applications, whereas SPR excitation requires incident light that is totally internally reflected. As a result, LSPR spectroscopy and imaging (LSPRi) are increasingly being integrated into live-cell microscopy experiments for the detection of secreted proteins while simultaneously imaging the cells with more traditional techniques such as fluorescence and bright field [6–9].

Continued progress in the field of nanoplasmonic biosensing is highly dependent upon meeting two design challenges related to surface functionalization. First, the reduced sensor area limits the number of conjugated ligands, making orientation critical for maximizing the probability of analyte capture. The most common ligands in biosensing are full antibodies, surface conjugated via their lysine residues or N-terminal amino groups. The typical antibody will have 60–80 of such residues resulting in a range of surface orientations, many of which are not optimal for analyte detection [10]. Second, experimental evidence and theoretical models show the nanostructures can exhibit a wide range of electromagnetic field decay lengths into the solution with some as short as 5 to 15 nm [11, 12]. Such nanoscale distances are largely filled when using intact IgG antibodies that have typical dimensions of 14.5 nm × 8.5 nm × 4.0 nm [13]. As a result, LSPR optimization requires at a minimum highly oriented full antibodies as ligands [14] and preferably lower molecular weight molecules which can be modified to enhance preferential orientation.

There are a number of classes of biomolecules which have been engineered with the goal of meeting these criteria including single chain antibodies (ScFvs), Fab fragments, and aptamers. Orientated ScFvs, which are roughly one fifth the size of full antibodies, were recently shown to enhance LSPR sensitivity relative to whole antibody counterparts [15]. In general, however, attempts to orient ScFvs and Fab fragments for improved biosensor sensitivity have produced decidedly mixed results [16–22, 10]. In addition, ScFvs and Fab fragments with molecular weights of approximately 27 and 50 kDa, respectively, are still relatively large ligands for many LSPR biosensing applications, leaving room for improved sensitivity if the size can be further reduced.

Single domain antibodies (sdAb), also called nanobodies, are the recombinant variable domain derived from heavy chain-only antibodies found in camelids, i.e., camels, llamas, and alpacas. Consisting of only a single domain endows sdAb with a number of attractive properties for biosensor applications. First, their small size (∼ 15 kDa or ∼ 1/10 the size of a full IgG) allows for facile production in yeast or bacteria, alone or as fusions with effector proteins. Second, both their small size and being a single domain provides them with remarkable thermostability, being able to refold and recover their binding ability following heat or chemical denaturation. Finally, sdAb are derived from true antibodies; thus, they possess the high affinity and specificity for which antibodies are renowned. SdAb, which recognize a plethora of targets, have been developed, and orientated sdAb have recently been demonstrated to improve SPR biosensor sensitivity [23, 24]. Our primary focus has been on the development of robust reagents for the detection of bio-threat agents [25, 26]. Of those reagents, the sdAb which bind ricin have been the most studied, and thus make well defined tools for testing the utility of sdAb in novel sensor applications [27, 28].

Here, we compare the sensitivity of LSPRi for orientated versus randomly surface-conjugated sdAb. Using anti-ricin sdAb, we first demonstrate the ability to modify the carboxyl terminus with positively charged peptide tails and rhizavidin fusion proteins for preferential surface orientation. The positively charged tail was designed to aid in surface orientation by means of an electrostatic interaction with a negatively charged surface. The rhizavidin fusion construct was designed to assist in orientation by binding to a biotinylated surface, preferentially directing the sdAb into the solution. Using SPR imaging (SPRi), we show that these modified sdAb have enhanced surface conjugation efficiencies and improved sensitivity to ricin relative to the non-orientated sdAb. We then measured the sdAb sensitivity to ricin on an LSPRi platform and compared the results to the biotin-neutravidin binding pair, a commonly used standard for biosensing applications and an apt comparison since biotin is readily orientated and ricin and neutravidin have similar molecular weights [3, 29]. Despite the fact that the sdAb ligands were 62-fold greater in mass than biotin, the measured sensitivities were statistically equivalent. We conclude that the relatively small size and the ability to readily modify sdAb for orientated surface conjugation make them optimal ligand candidates for LSPR imaging and spectroscopy applications. The current sensitivity study lays the groundwork for future work in determining the LSPRi limit of detection of each construct and multiplexed toxin-sensing applications.

## Experimental

The sequence and binding characteristics of the ricin-binding sdAb C8 and D12 have been previously described [27, 28]; both have sub-nM affinity for the toxin ricin. The clone D12f is a version of D12 in which an unpaired cysteine was mutated to a serine causing no change in sdAb affinity. The C8 sdAb, lacking the upper hinge region, was cloned into pET22b for protein expression. Protein was purified using immobilized metal affinity chromatography followed by size exclusion chromatography on an FPLC as previously described [26, 30]. Protein sequences of all sdAb constructs are detailed in the Supplementary Material.

The construct referred to herein as C8-zip is a genetic fusion of the C8 sdAb with a positively charged leucine zipper described by Oshea et al. [31]. DNA including a glycine-serine linker followed by the zipper sequences was synthesized by GenScript (Piscataway, NJ) and included flanking Not I and Xho I restriction endonuclease sites. We cloned the linker-zipper sequence into pET22b and then mobilized the C8 sdAb into the modified pET22b through the Nco I and Not I sites; DNA sequencing was used to confirm the constructs. Protein was produced from the periplasmic space in a protocol identical to that used to purify C8. This C8-Zip construct had initially been prepared to facilitate sdAb heterodimer formation [32].

The D12f-rhiz construct is a genetic fusion of the D12f sdAb with the biotin binding protein rhizavidin (rhiz). Unlike our previously described sdAb-rhiz fusion construct, the D12f-rhiz does not include the upper hinge sequence between the D12f and rhiz. Protein production was as described for our previous sdAb-rhiz fusions [33].

Details of the LSPR chip fabrication and the LSPRi measurement setup have been published previously [34, 29]. In short, square arrays of gold nanostructures were patterned on No. 1.5 glass coverslips using electron-beam nanolithography (Raith GmbH) to expose a bilayer resist structure consisting of polymethyl methacrylate and ethyl lactate methyl methacrylate copolymer purchased from Microchem Corp. Each array measured 6 μm × 6 μm and consisted of 400 evenly spaced nanostructures separated by a pitch of 300 nm. The bases of the nanostructures were circular in cross section with diameters of 70 ± 5 nm and the heights were 75 ± 2 nm, giving a plasmonic resonance peak centered at ∼ 635 nm in 10 mM phosphate buffered saline (Thermo Scientific). Protein binding to the Au surface creates a perturbation in the local index of refraction which induces a redshift in the resonance peak as well as enhanced scattering. In the LSPRi measurement, this response is manifested as an increase in the nanoplasmonic array’s brightness as imaged by the camera. We have shown, using analytes such as neutravidin and IgG proteins, that this response can be calibrated to the fractional occupancy of surface-bound receptors. Commercial SPRi has sensors that are orders of magnitude larger in surface area but operates on a similar physical principle. When using the same functionalization chemistries, results from the two techniques can be correlated [8, 34].

The chip was loaded onto a custom-built microfluidic assembly consisting of a 300-μL chamber to which the analyte solution was introduced using a peristaltic pump from Instech Laboratories (P720). All imagery was acquired using Zeiss Zen software, an inverted Zeiss Axio Observer microscope, a 40X/1.4 NA objective, and a thermoelectrically cooled 16-bit sCMOS camera from Hamamatsu (Flash 4.0) operated in 2 × 2 binning mode. Images were collected in a reflected light geometry using Koehler illumination, a 100 W halogen lamp, and crossed polarizers to reduce the collection of light scattered from the substrate. Error bars in the LSPRi response represent the standard deviation from nine averaged arrays. Unless otherwise shown, the error bars are within the size of the data markers.

SPRi measurements were conducted with Bio-Rad’s XPR36 protein analysis system, the sensors of which consisted of a gold thin film deposited atop a glass prism for excitation of surface plasmon polaritons by total internal reflection. Once inserted into the instrument, each chip is microfluidically arrayed into 36 measurement spots. This enabled a range of sdAb ligands to be functionalized on a single chip in contrast to the LSPRi setup in which each experiment consisted of only one ligand type. All LSPRi and SPRi measurements were conducted at 25 °C. Error bars in the SPR response are the standard deviation from averaging five measurement spots on the same chip. Unless otherwise shown, the error bars are within the size of the data markers.

For SPRi studies of sdAb, the Bio-Rad chips were cleaned down to the bare gold surface by RF plasma ashing at 40 W in 300 mTorr of a 5 % hydrogen, 95 % argon mixture, and then functionalized with a two-component self-assembled monolayer (SAM) by immersion for 18 h in an ethanolic-based thiol solution (0.5 mM), consisting of a 3:1 ratio of SH-(CH_2_)_8_-EG_3_-OH (SPO) to SH-(CH_2_)_11_-EG_3_-COOH (SPC) (Prochimia Surfaces Sp.) for C8 and C8-zip studies. The chips were then rinsed with EtOH and dried under flowing nitrogen gas. Activation of the SPC component consisted of introducing a 33 mM: 133 mM ratio of N-hydroxysulfosuccinimide (sulfo-NHS) and 1-ethyl-3-[3-dimethylaminopropyl]carbodiimide hydrochloride (EDC) from Thermo Scientific in ultrapure water (DDW; EMD Millipore) for 5 min at a flow rate of 30 μL/min. This was followed by a 5-min rinse with DDW and the introduction of the sdAb under the range of concentration and pH conditions described in the “Results and Discussion” section. For the D12f-rhiz studies, a 2-mg/mL solution of (+)-biotinyl-3,6-dioxaoctanediamine (amine-PEG_2_-biotin) from Thermo Scientific was introduced over the activated surface at 30 μL/min for 5 min followed by a range of D12f-rhiz concentrations as described in the “Results and Discussion” and Supplementary Material sections. Finally, in all cases, unreacted –COOH groups were blocked by flowing 0.1 M ethanolamine (Bio-Rad) in PBS for 5 min at 30 μL/min. For the LSPR chips, this process was repeated except all solutions were manually drop coated and washed while inside the custom-built microfluidic assembly.

The identical surface cleaning and SAM layer formation protocol was applied for LSPRi biotin-neutravidin studies except that the two-component SAM layer consisted of a 3:1 ratio of SH-(CH_2_)_8_-EG_3_-OH (SPO) to SH-(CH_2_)_11_-EG_3_-NH_2_ (SPN) (Prochimia Surfaces Sp.). LSPR chips were drop coated with 100 μL of 2 mg/mL sulfo-NHS-biotin (Thermo Scientific) in PBS, incubated for 30 min at room temperature, rinsed with DDW, and dried in flowing nitrogen.

## Results and Discussion

Three ligands were used in this study: an unmodified sdAb (C8), a genetic fusion of C8 with a positively charged peptide (C8-zip), and a genetic fusion of the D12f sdAb with the biotin binding protein rhizavidin (D12f-rhiz). Both the C8 and D12f sdAb that compose the ligand provide the recognition function of the constructs and are specific for ricin. They bind the same epitope on the toxin and exhibit nearly identically sub-nM affinities for their cognate antigen. Structurally, the binding loops of sdAb are located on the opposite side from their C-terminus, enabling the construction of fusions to facilitate directional immobilization. We hypothesized that the positively charged lysines on the C-terminus of C8-zip protein would maximize the potential for directional immobilization on the negatively charged –COOH surface, resulting in an orientation with the tail coupled to the surface and the binding region oriented into solution for optimal antigen capture. In a similar way, the D12f-rhiz enables directional immobilization but via the biotinylated surface [33].

**Fig. 1:**
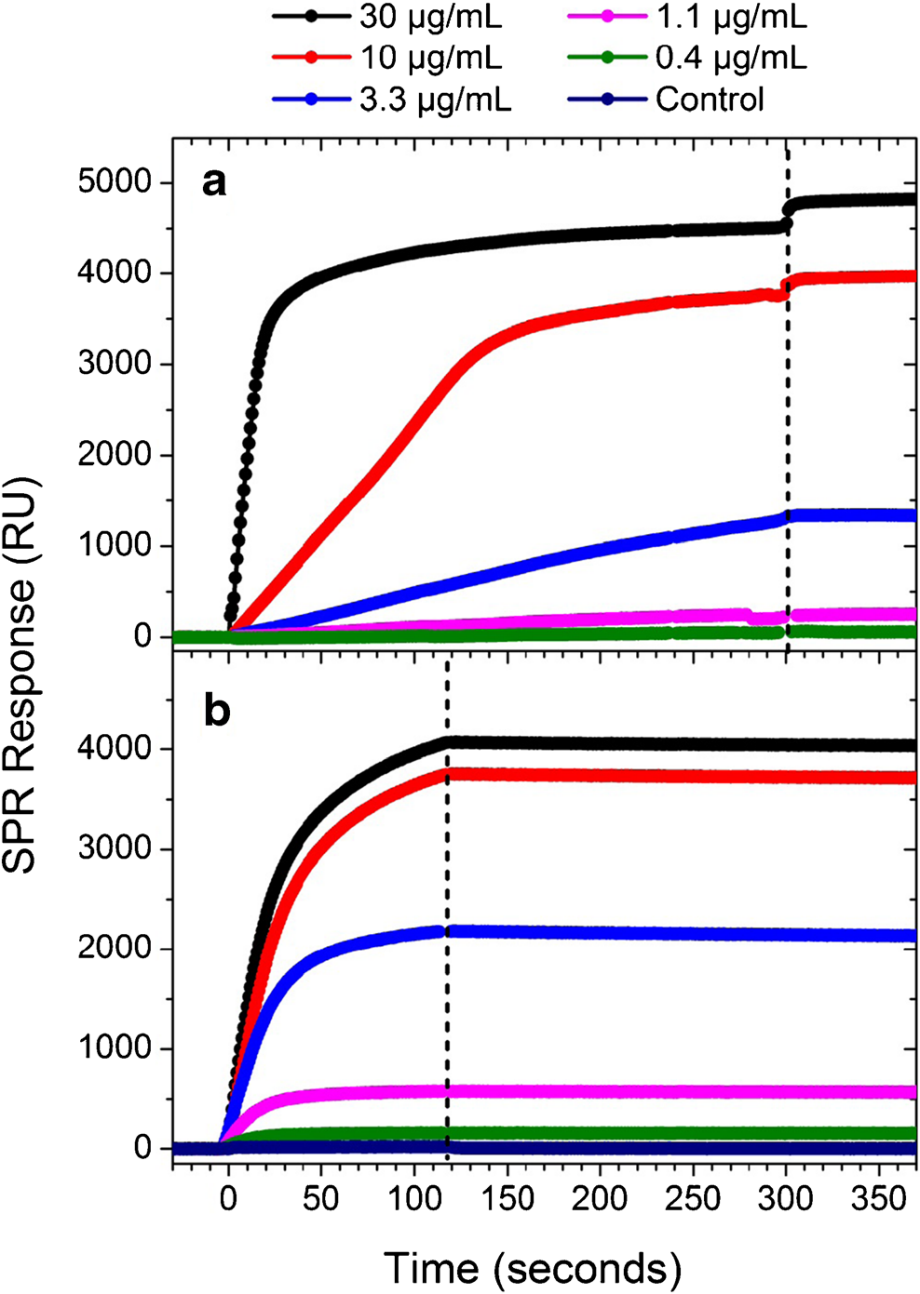
**a** SPR response to the conjugation of C8-zip sdAbs for a range of ligand concentrations followed by the introduction of 100 nM ricin in (**b**). The control study in **b** consisted of a 100-nM ricin solution incubated with 5 μM of C8 for 45 min to block the binding sites. It was then introduced over a 4.0-μg/mL C8 functionalized surface. The vertical *dashed line* separates the association phase (*left*) in which the ligand or analyte solution is flowing over the surface from the dissociation phase (*right*) in which buffer flows over the surface

To optimize the plasmonic sensitivity to ricin, a range of ligand concentrations were introduced over the EDC/Sulfo-NHS activated SPR surface followed by a saturating concentration of ricin (100 nM). Fig. 1a shows the SPR response to C8-zip in which the concentration was varied from 0.4 to 30 μg/mL in PBS, pH 7.0. At the higher ligand concentrations, there were diminishing returns with regards to ricin-binding capacity (Fig. 1b) with the 30 μg/mL exposed surface binding only 8 % more than the surface coated at 10 μg/mL. Exceeding this 30 μg/mL value resulted in a rapid increase in non-specific binding and minimal ricin sensitivity enhancement. C8 and D12f-rhiz exhibited similar surface saturation concentrations, lying between 1 and 30 μg/mL (Supplementary Material). Ligand concentration values such as the 30 μg/mL value for C8-zip were defined as optimal based on these criteria of maximizing sensitivity and minimizing non-specific binding. A control study was also conducted in which a 100-nM ricin solution was first incubated with 5 μM of C8 for 45 min to block the ricin-binding sites. When the blocked ricin was introduced over a C8-functionalized surface, no response was detectable, demonstrating levels of non-specific binding below the SPRi limit of detection (Fig. 1b).

**Fig. 2:**
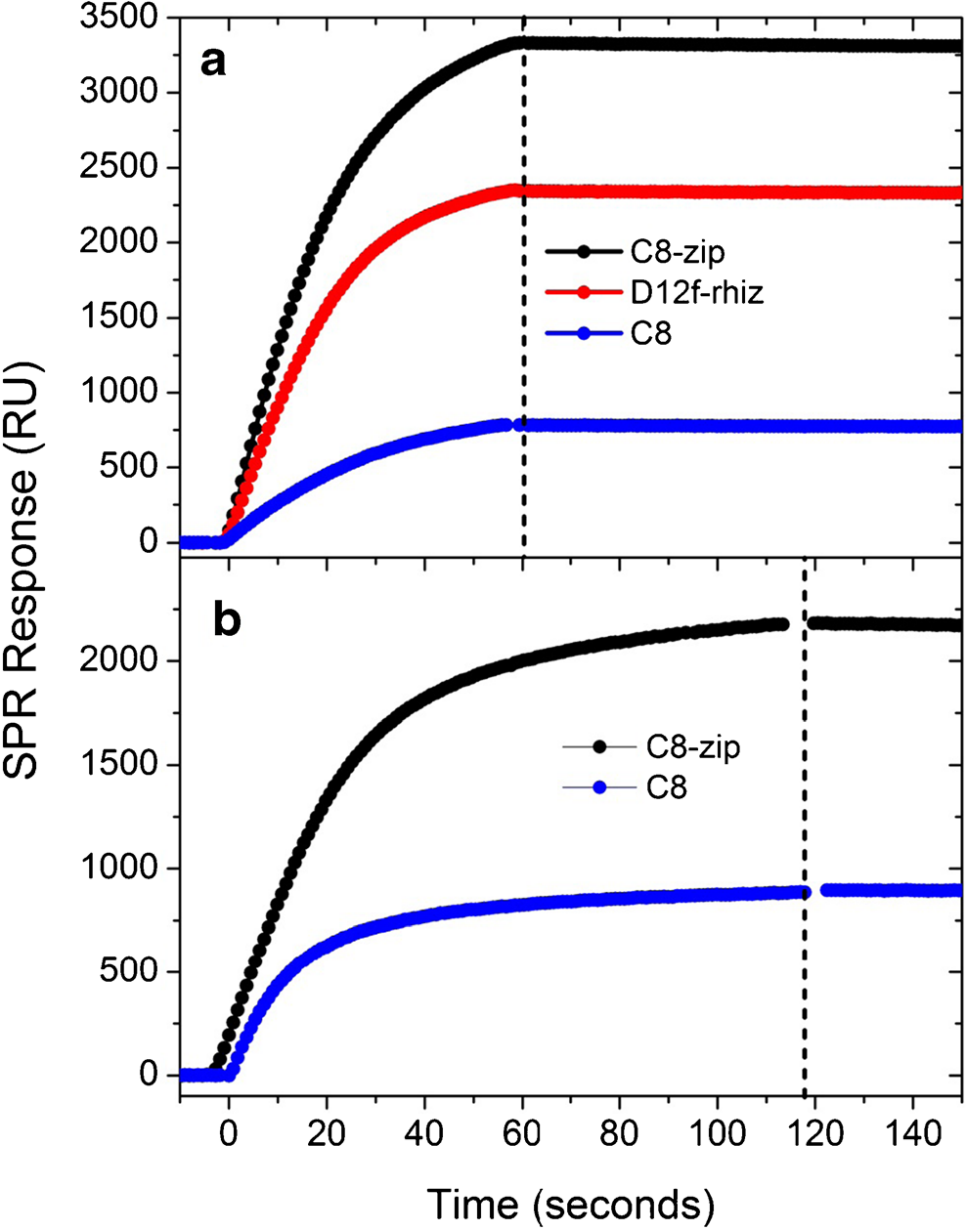
**a** SPR response to 100 nM ricin of optimally prepared C8-zip, C8, and D12f-rhiz surfaces. **b** SPR analyte response to 100 nM ricin following C8-zip and C8 ligand conjugations with responses of 1300 ± 120 RU. Measurements were conducted in parallel by multiplexing a single sensor chip to eliminate chip-to-chip variations. The vertical *dashed line* separates the association phase (*left*) in which the analyte solution is flowing over the surface from the dissociation phase (*right*) in which buffer flows over the surface

A study was then conducted of SPR sensitivity to ricin at each ligand’s optimal concentration. The 36-array microfluidic architecture of the XPR36 allowed for these measurements to be taken simultaneously alongside control lanes, thus eliminating uncertainties from chip-to-chip variations. C8-zip exhibited the highest response to 100 nM ricin, 320 % higher than that of C8 and 42 % higher than that of D12f-rhiz (Fig. 2a). In order to help isolate sensitivity improvements due to ligand orientation, we next introduced ricin over C8 and C8-zip surfaces prepared with similar ligand responses (1300 ± 120 RU) which is indicative of equivalent ligand surface densities. Figure 2b shows the SPR response to 100 nM ricin for these surface conjugations. The C8-zip surface again exhibited the highest response to 100 nM ricin, 140 % higher than that of C8.

To further investigate relative ligand orientation, we compared the analyte binding activity, *A*, of the conjugated C8 and C8-zip ligands, defined as the moles of captured analyte divided by the moles of surface-conjugated ligand. In SPR, the moles of a given molecule deposited on the surface is proportional to the instrument response, *X*, divided by the molecular weight, *m*, so that the activity is readily expressed as

$$ A=\frac{X_A\cdot {m}_L}{X_L\cdot {m}_A} $$

where *X*_*L*_ and *X*_*A*_ are the SPR responses in RU to the binding of ligand and analyte, respectively, *m*_*L*_ and *m*_*A*_ are the molecular weights of the ligand and analyte, respectively, and it is assumed that the ligands are monovalent.

**Fig. 3:**
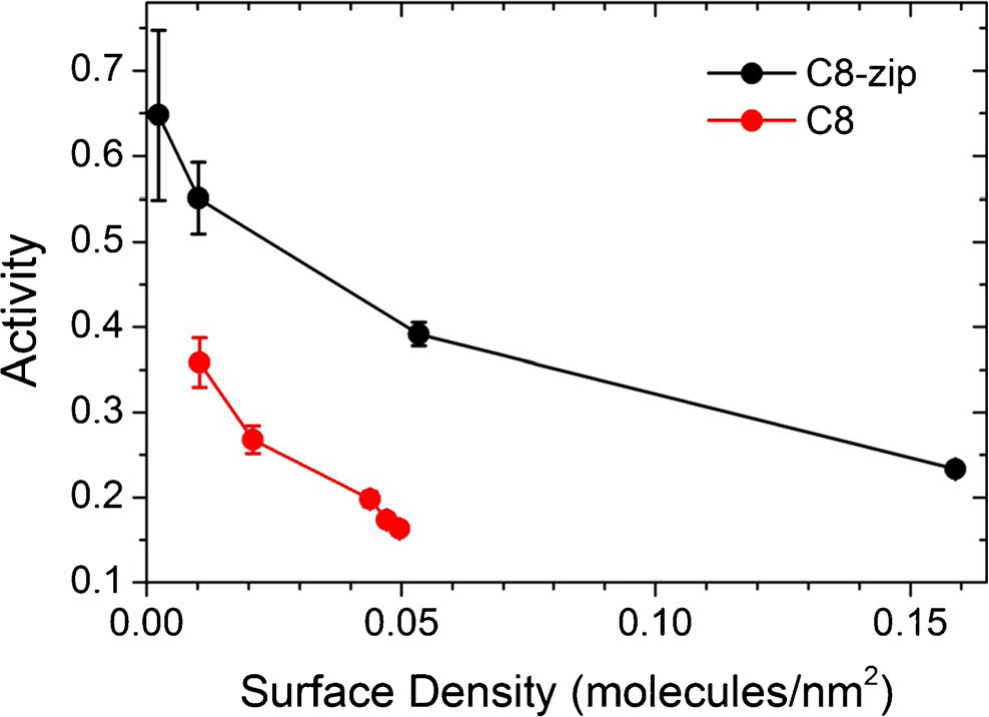
Ricin saturation activity versus ligand surface density for C8-zip and C8. Ligand surface density was calculated assuming a sensor calibration of 1 RU = 1 pg/mm^2^. The ricin concentration was 100 nM for all experiments

To properly isolate the effect of orientation on *A*, care must be taken to work in the dilute ligand limit in order to minimize steric hindrance effects. If analyte molecules can repel one another at the surface, the result is a reduced activity value unrelated to ligand orientation. Figure 3 compares the activity of C8-zip to C8 for a range of surface ligand densities by exposing the surface to ligand concentrations of 0.25 to 10 μg/mL for 300 s. The ligand surface density was calculated assuming a sensor calibration of 1 RU = 1 pg/mm^2^ [35]. At higher surface densities, the activity values of the two ligands converge as expected for a surface dominated by steric hindrance. As the surface concentration is reduced, both exhibit marked increases with maximum C8-zip and C8 activities of 65 and 36 %, respectively. The results indicate that the positively charged tail of the C8-zip improved surface orientation and are consistent with the enhanced sensitivity measured in Fig. 2. We also observed improved sensitivity for the orientated sdAb when using SPR GLC sensor chips, designed by Bio-Rad for general amine coupling via a compact polymer layer with a binding capacity of approximately one protein monolayer (Supplementary Material).

**Fig. 4:**
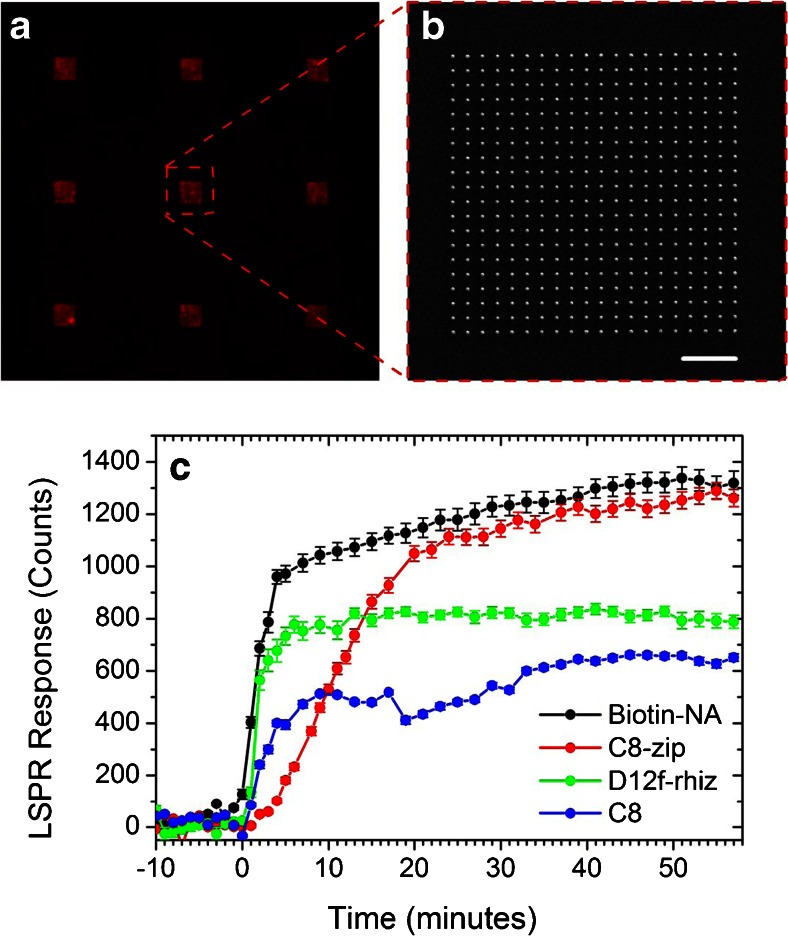
**a** LSPRi of nine arrays, each array consisting of 400 nanostructures. The image is false colored *red* to indicate the resonance wavelength of 635 nm. **b** Scanning electron microscopy of an array of 400 nanostructures. **c** LSPRi response to 100 nM ricin for C8-zip, C8, and D12f-rhiz surfaces compared to that of a biotinylated surface for 100 nM neutravidin

For the corresponding nanoplasmonic response studies, a functionalization protocol identical to that used for the SPR study in Fig. 2a was applied to the gold nanostructures of the custom-made LSPR chips. Response data to 100 nM ricin were determined by averaging the mean intensity of nine arrays, each array consisting of 400 nanostructures (Fig. 4a, b). As with the SPR results, the C8-zip surface was the most sensitive of the three ligands, with a saturation response that was 104 % greater than that of C8 and 61 % greater than that of D12f-rhiz (Fig. 4c).

To further characterize the sensitivity of the C8-zip functionalized surface, we conducted LSPRi studies in which the surface was functionalized with biotin and exposed to a 100-nM neutravidin solution, a commonly used standard in nanosensor characterization. Both of these receptor-ligand pairs have exceptionally high binding affinities and both ricin and neutravidin have approximately the same molecular weight (60 kDa). Biotin, however, is readily orientated and has a molecular weight of 244 Da, 62 times lower than that of C8-zip. Thus, the differences in LSPRi sensitivity to analyte should be reflective of ligand size and degree of orientation. Interestingly, Fig. 4c shows the responses of the C8-zip and biotinylated surfaces to their cognate analytes were statistically equivalent, demonstrating that the small size and highly oriented conjugation of the C8-zip molecules are well suited for LSPRi applications. Future work will build on these results to estimate the limit of detection for each construct.

## Conclusions

When conjugated to plasmonic nanostructures, orientated sdAb exhibited enhanced LSPRi sensitivity which matched that of biotinylated surfaces to neutravidin, long considered a model system for small and orientated ligand studies. The additional facts that sdAb produce well in bacteria and yeast, are readily modified, and robust to denaturation make them well suited for a broad range of SPR and LSPR biosensing applications. The application of LSPRi to ricin detection is an important step forward in the design of small, readily deployable biosensors against bio-threat agents.

## Electronic Supplementary Material

ESM 1 (DOCX 620 kb)
