# Supplementary material for: Optimizing Nanoplasmonic Biosensor Sensitivity with Orientated Single Domain Antibodies
Source: Plasmonics. 2015 May 26;10(6):1649–55. doi: 10.1007/s11468-015-9969-3 (PMC4644190; doi:10.1007/s11468-015-9969-3)
Supplement: Supplementary file 1 — (DOCX 620 kb) [file 11468_2015_9969_MOESM1_ESM.docx]

*Optimizing Nanoplasmonic Biosensor Sensitivity with Orientated Single Domain Antibodies*

Supporting Information

S1. Ligand Saturation Studies

Ligand conjugation studies for C8 and D12f-rhiz are shown in Figures S1 and S2. At concentrations above 1 ug/mL the C8 showed signs of non-specific binding as evidenced by the negative slope of the response curve after 150 s of association and continuing through the dissociation phase. Non-specific binding of D12f-rhiz increased for concentrations above 3 ug/mL as can be seen by the negative slope in the dissociation phase (Figure S2). Increasing amounts of non-specific binding during the ligand conjugation step coupled with diminishing returns with respect to ricin sensitivity were the criteria used to determine optimal ligand conjugation conditions.


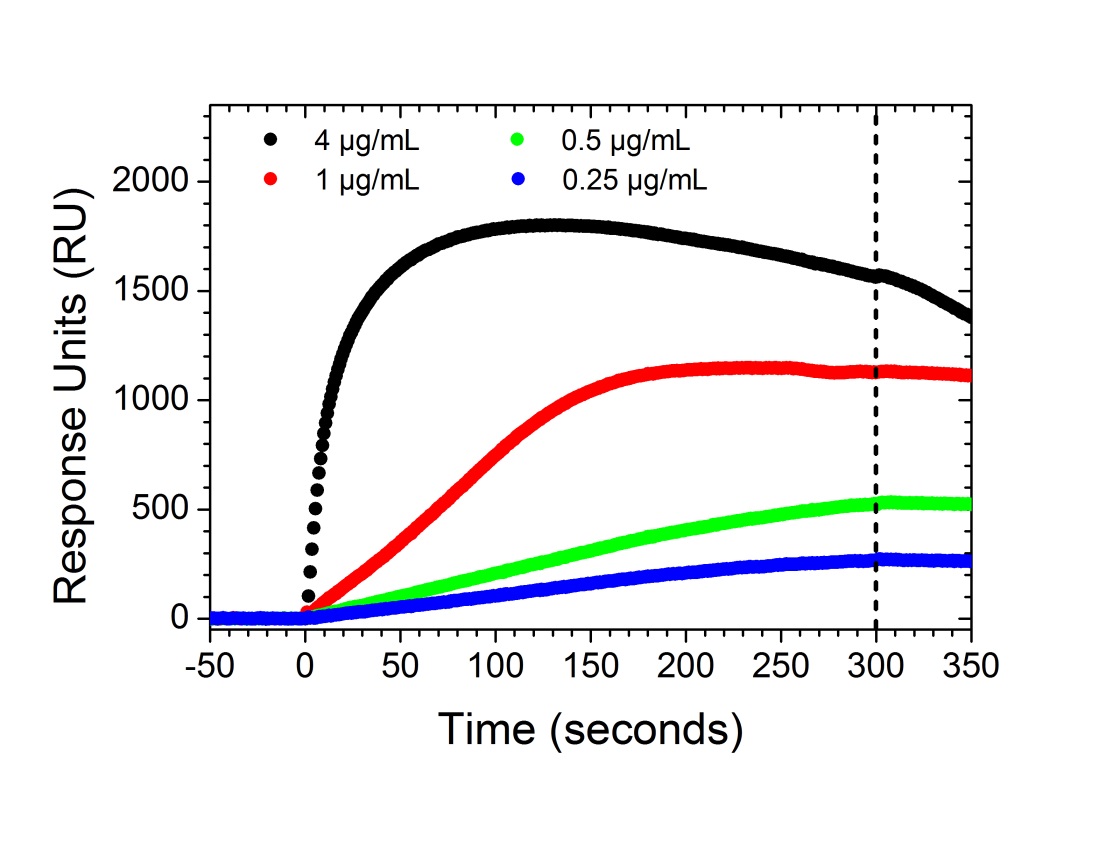


Fig S1. SPR response to the conjugation of C8 for a range of ligand concentrations. The vertical dashed line separates the association phase (left) in which the ligand or analyte solution is flowing over the surface from the dissociation phase (right) in which buffer flows over the surface.


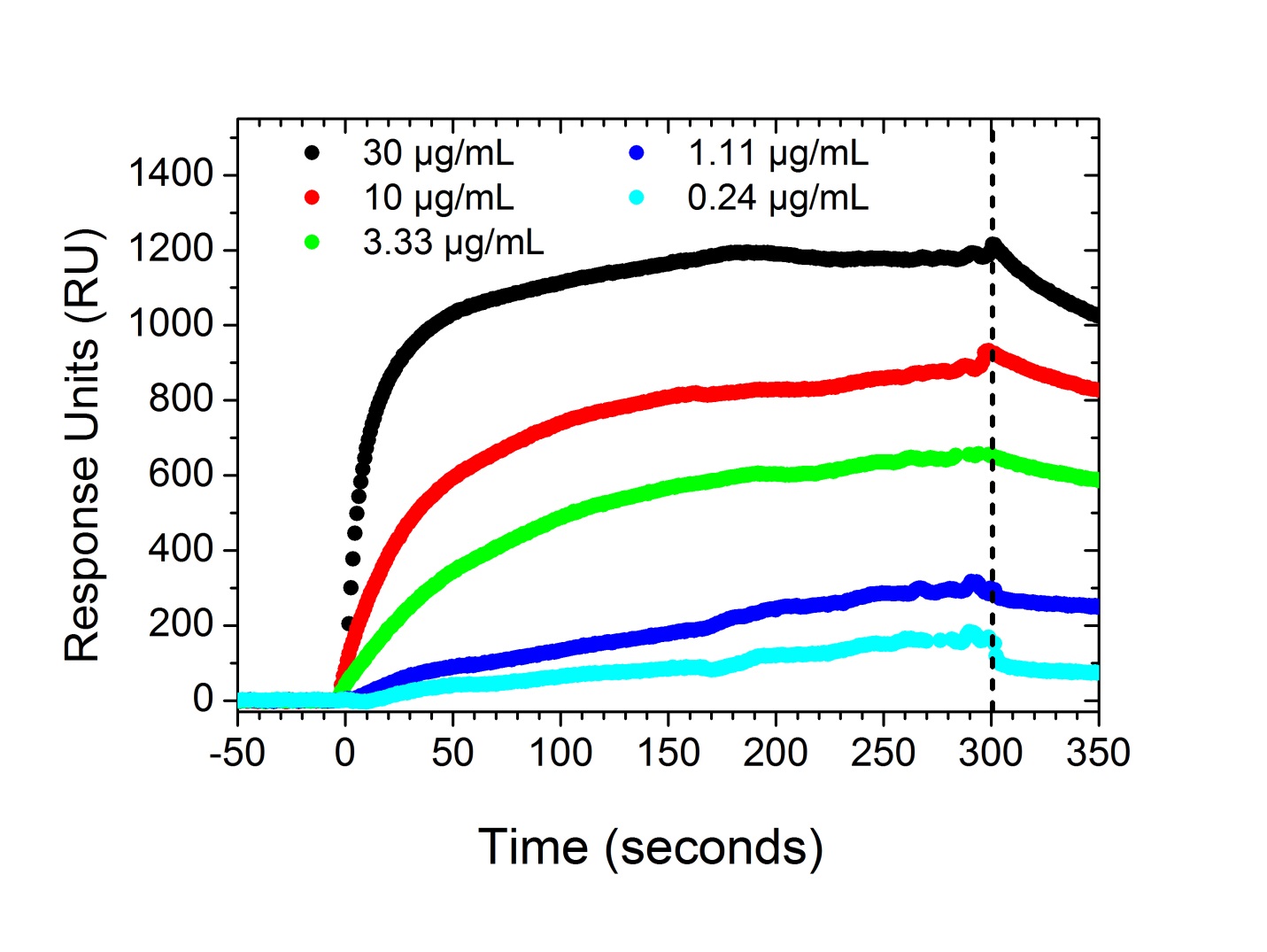


Fig S2. SPR response to the conjugation of D12f-rhiz sdAbs for a range of ligand concentrations. The vertical dashed line separates the association phase (left) in which the ligand or analyte solution is flowing over the surface from the dissociation phase (right) in which buffer flows over the surface.

S2. GLC Chip Sensitivity to Ricin and Ricin A for C8 and C8-zip Surface Conjugations

The improved sensitivity of the orientated C8-zip sdAb versus unorientated C8 was also validated on the Bio-Rad XPR36 using commercially available GLC chips. These chips consist of a compact polymer layer for general amine coupling with a binding capacity of approximately one protein monolayer. The association phase of 100 nM ricin binding to C8-zip and C8 functionalized surfaces is shown in Figure S3. The sdAbs were surface conjugated using the same EDC/sulfo-NHS coupling protocol as for the two-component SAM surfaces. The sdAb concentration was μg/mL, introduced at a flow rate of 30 μL/min for five min. After 60 seconds the C8-zip response was 67% greater than C8, a clear improvement though diminished from what was observed for the two component SAM layer discussed in the main text (320%). For completeness the response to 33 nM of the ricin A chain was investigated in parallel and similar relative responses were observed (Figure S4).


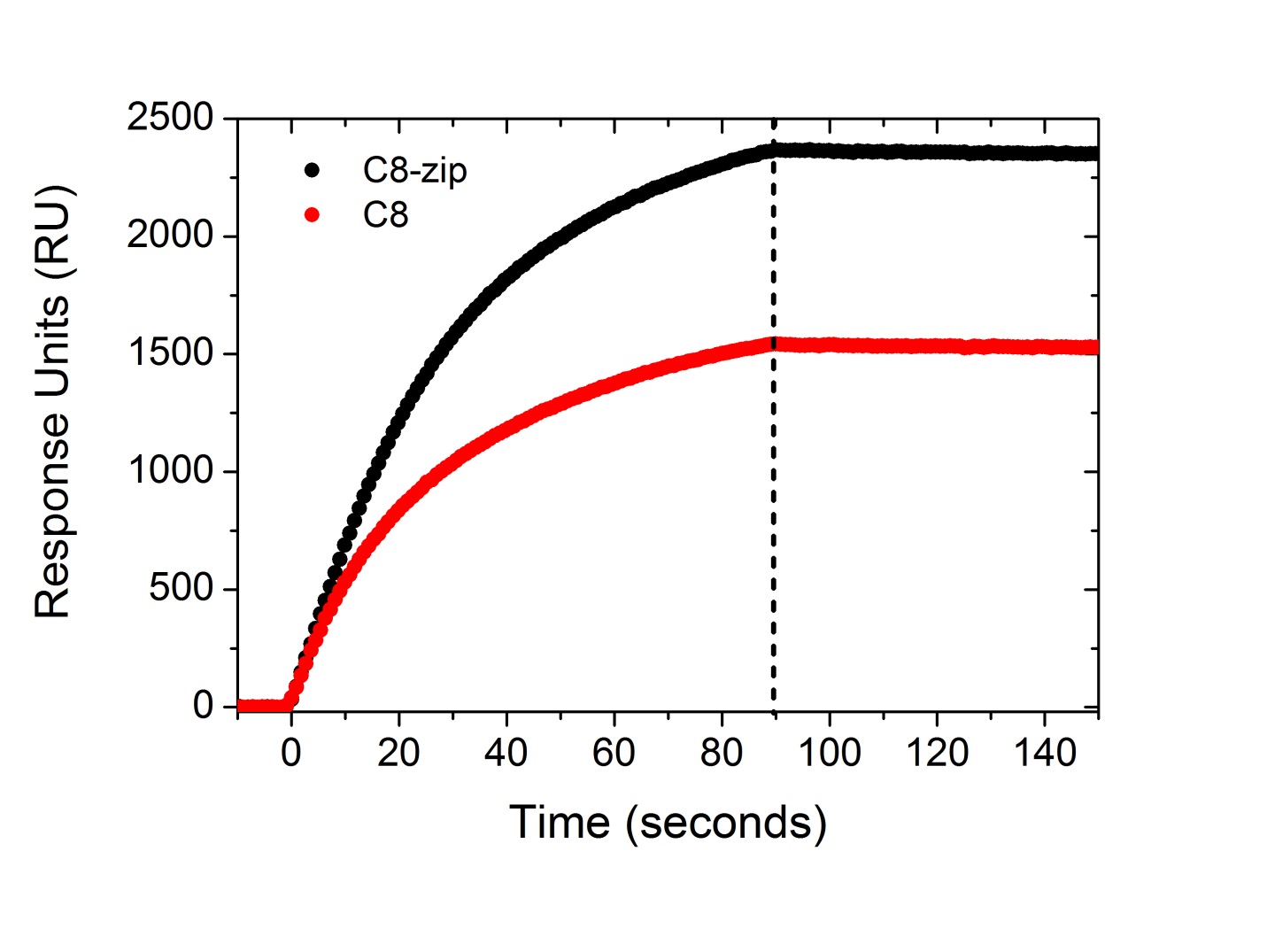


Figure S3. SPR Response of optimally prepared C8-zip (black) and C8 (red) surfaces to 100 nM ricin. The vertical dashed line separates the association phase (left) in which the ligand or analyte solution is flowing over the surface from the dissociation phase (right) in which buffer flows over the surface.


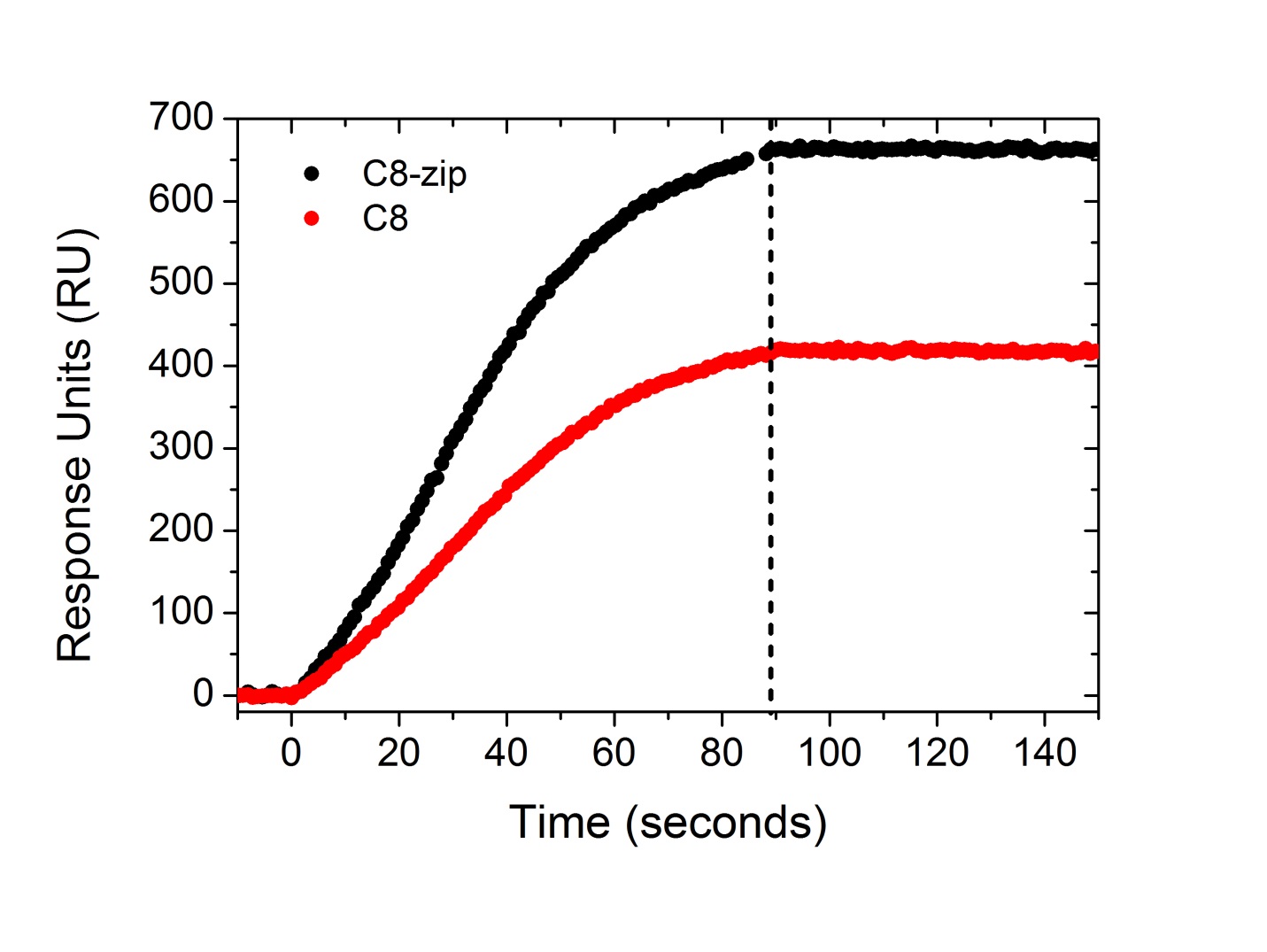


Figure S4. SPR Response of optimally prepared C8-zip (black) and C8 (red) surfaces to 33 nM ricin A. The vertical dashed line separates the association phase (left) in which the ligand or analyte solution is flowing over the surface from the dissociation phase (right) in which buffer flows over the surface.

S3. Sequence of C8, C8 zip and D12f-rhiz in single letter amino acid code

C8 Sequence

EVQLQASGGGLVQGGDSLRLSCAASGRTLGDYGVAWFRQAPGKEREFVSVISRSTIITDYANSVKGRFTISRDNAKNAVYLQMNSLKPEDTAVYYCAVIANPVYATSRNSDDYGHWGQGTQVTVSSAAALEHHHHHH

C8-zip Sequence

EVQLQASGGGLVQGGDSLRLSCAASGRTLGDYGVAWFRQAPGKEREFVSVISRSTIITDYANSVKGRFTISRDNAKNAVYLQ**M**NSLKPEDTAVYYCAVIANPVYATSRNSDDYGHWGQGTQVTVSSEPKTPKPQPAASGAEFAAAGGGGSGGGGSGGGGSAQLKKKLQALKKKNAQLKWKLQALKKKLAQGGDALEHHHHHH

D12f-rhiz Sequence

EVQLQASGGGLVQDGGSLRLSCAVAGRPLSDYGVGWFRQASGKEREFVAVISGSGIVTDYANSVKGRFTISRDVAKNVVHLQMNSLKPEDTAVYYCAALTNPVYAASRNSNDYGYWGQGTQVTVSSAAALEFDASNFKDFSSIASASSSWQNQSGSTMIIQVDSFGNVSGQYVNRAQGTGCQNSPYPLTGRVNGTFIAFSVGWNNSTENCNSATGWTGYAQVNGNNTEI

VTSWNLAYEGGSGPAIEQGQDTFQYVPTTENKSLLKDLEHHHHHH
